# *In-situ* synthesized and pattern Ag/Bi_2_Se_3_ composite structure by LDW and photothermal conversion

**DOI:** 10.1038/s41598-019-38496-9

**Published:** 2019-02-11

**Authors:** Zejia Zhao, Guozhi Jia, Yanling Liu, Qiurui Zhang, Yaoyao Zhou

**Affiliations:** Tianjin Chengjian University, Tianjin, 300384 China

## Abstract

Bi_2_Se_3_ nanofilm has exhibited many promising potentials application in the field of photo-to-heat conversion. A highly-efficient photo-to-heat conversion system of Ag/Bi_2_Se_3_ composite nanofilm is successfully fabricated through laser direct writing (LDW) technique. The localized heat induced by laser simultaneously achieve Ag particles synthesis, transfer and patterning in a single processing step. The thermal reaction process includes the forming of nanoparticles based on the process of the thermal reduction, laser ablation, sputtering deposition and so on. The thermal storage capability and photothermal conversion stability have been greatly improved through preventing the heat from loss and efficient LSPR enhancing. The photothermal conversion mechanism of composition film is discussed in detail. This work suggests that the laser-assisted transfer technique give rise to a new expectation of functional composite nanofilm application for energy conversion.

## Introduction

Photo-to-heat conversion performance of nanomaterials has been in the spotlight for new energy applications due to the basic demand for energy harvesting^1,2^. Photothermal conversion in the UV-visible-NIR range is well reported in nanomaterial dispersions in various solvents or polymer matrices, upon exposure to a laser source^3^. The effect of heat generation offered by these nanoheaters has been applied in a variety of researching areas such as biological imaging, hyperthermal cancer therapy, drug delivery, photocatalysis, study of phase transitions and water evaporation^4–10^.

The photothermal conversion coefficient plays a critical role in efficient use of the photo-to-heat energy^11,12^. The narrow bandgap semiconducting materials^13^, especially for topological insulator Bi_2_Se_3_ with rhombohedral crystal structure, has intrinsic fundamental simple band structure near the Dirac point and exhibit very high light absorption coefficients for a broad range of wavelengths^14–16^. Owing to the unique electronic structures, resulting in a great deal of potential applications including photonics, spintronic and photothermal^15,17–20^. Currently, a great deal of investigations have been reported on combining Bi_2_Se_3_ nanostructure with other nanomaterials, such as CdSe/Bi_2_Se_3_ quantum dots prepared by action exchange reaction^12^, the photothermal conversion ability can be extremely improved due to the transport path of carriers changed based on the energy band theory. Likewise, surface-bound collective excitations of free carriers existing in noble metals can be excited by light, which can cause resonance phenomenon in the driving electromagnetic field. Because of the enhanced near-surface electric fields, so-called localized surface plasmon resonances (LSPR), the nanocrystals show intense light absorption and scattering^21^. These properties can contribute to an extensive application, including pyroelectricity and photothermal conversion.

Ag nanoparticles (NPs) as an important LSPR material is with foreground application in various fields due to large scattering cross-sections and relatively large molar extinction coefficients^22,23^. Many studies have shown that incorporation of Ag atom into the Bi_2_Se_3_ films plays a significant role in optical and optoelectronics aspect via the theory and experiments investigation^24,25^. A variety of techniques have been reported to prepare Ag-doped nanomaterials thin films in the literature such as thermal evaporation, photochemical synthesis and photocatalytic activity, it can be still necessary to develop another method to prepare the composition structure materials for further applications. The LDW transferring nanotechnology has been of great interest for the fabrication of nanomaterials electronic devices for potential applications including sensors, transparent electrodes and so on^26,27^. The technique exhibits high through-put and parallel processes to design flexibility at a low cost. The most prominent characteristic is that the laser cannot cause significant problems for gradually phase converted by heat and light over time. The nanomaterials without being acted by laser can be removed, while laser acting pattern area can be transferred to the receiving substrate.

Here, we put forward the idea of creating Ag/Bi_2_Se_3_ nanocomposites based on the silver nanocrystal synthesizing, transfer and pattern technology simultaneously. The composite materials are successfully achieved via LDW technology^28,29^. The method can transfer and pattern different nanomaterials to the various substrate through a single-step process. The photothermal characteristic experiments manifest Ag/Bi_2_Se_3_ nanocomposites possess strong photothermal conversion capacity and excellent heat stability. This research provides a new path to prepare the composition nanofilms in the aspect of photo-to-heat application by LDW technology.

## Results

**Figure 1:**
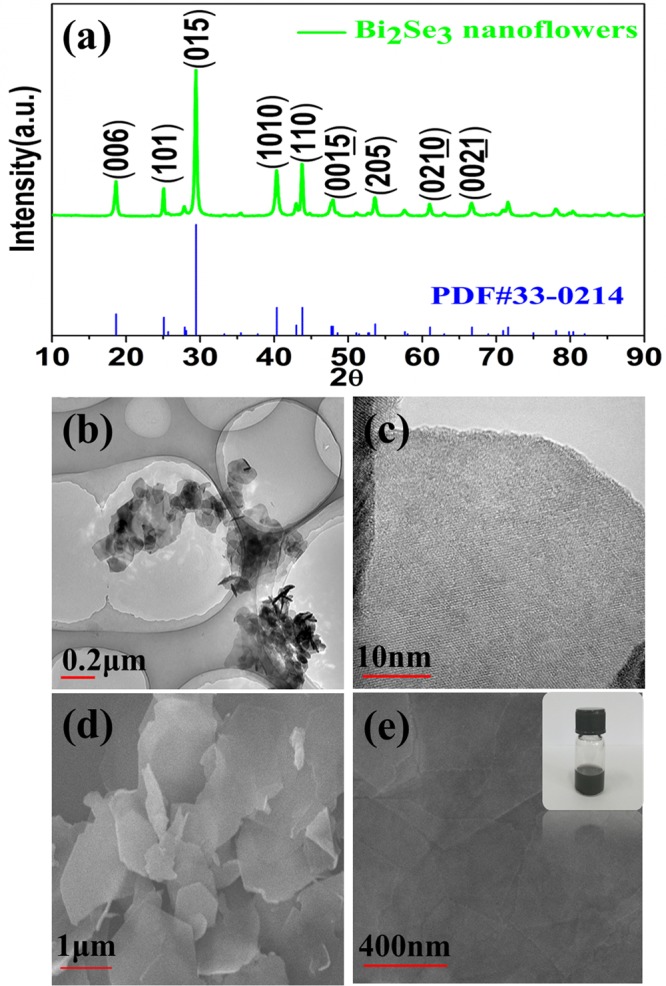
(**a**) XRD pattern of the sample of Bi_2_Se_3_. (**b**) Typical TEM image of Bi_2_Se_3_ nanosheets. (**c**) Typical HRTEM image of Bi_2_Se_3_ nanosheets. (**d**) SEM images of Bi_2_Se_3_ nanosheet samples. (**e**) Typical SEM image of Bi_2_Se_3_ nanosheets and the figure inset shows the photograph of Bi_2_Se_3_ nanosheet ethanol solution.

The morphology and composition structure of the as-prepared samples are analyzed by TEM, SEM and XRD, as showed in Fig. 1. Figure 1(a) illustrates XRD patterns of Bi_2_Se_3_ determine the composition and structure of the sample synthesized by microwave-assisted method. The main diffraction peaks can be readily indexed into the rhombohedral phase of Bi_2_Se_3_, which is well agreement with the JCPDS Card No. 33–0214. The well-defined peaks in XRD pattern indicate the forming of high quality Bi_2_Se_3_. The microstructure characteristics of the as-prepared Bi_2_Se_3_ samples are analysed by transmission electron microscopy (TEM), which shows that Bi_2_Se_3_ nanosheets have several hundred nanometers in lateral size in Fig. 1(b). High-resolution TEM (HRTEM) images show that the nanosheets are consisted of numerous Bi_2_Se_3_ nanocrystals with a high-quality crystal. The morphology of the as-prepared Bi_2_Se_3_ is characterized by SEM and is shown in Fig. 1(d,e), the results indicate that the sample consisted of well-defined hexagonal nanosheets with smooth surfaces. And, from the insert Fig. 1(e), it is clearly seen that the ethanol solution of Bi_2_Se_3_ nanosheets is pretty stable owing to the low interfacial energy between ethanol solvent and Bi_2_Se_3_ nanosheets^30,31^.

**Figure 2:**
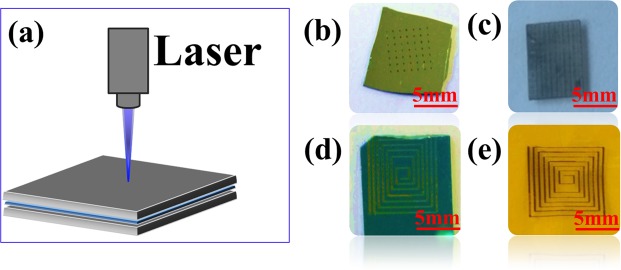
(**a**) Schematicdiagram of the laser direct-writing selective area transfer patterning of Bi_2_Se_3_. (**b**) Images of Bi_2_Se_3_ pattern on the silicon substrate (A square pattern consisting of dots). (**c**) Laser-treated Bi_2_Se_3_ film (square pattern) located between glass substrate before. (**d**) Bi_2_Se_3_ pattern on the Si/SiO_2_ substrate. (**e**) The images of Bi_2_Se_3_ patterns on Polyimide (PI) film.

Preparing and patterns of Ag/Bi_2_Se_3_ composition film by LDW is shown in Fig. 2. Figure 2(a) illustrates the schematic of the fabrication process for Bi_2_Se_3_ pattern on various substrates by the laser direct writing technique. Under the circumstances, transfer proceeded, by the laser-induced, resembling ejection of a “flyer” that is projected away from the donor film and toward the receiving substrate, where it landed, causing the formation of a voxel. Laser-treated Bi_2_Se_3_ film (square pattern) located between before. Digital images of Bi_2_Se_3_ pattern on the silicon substrate, glass substrate, Si/SiO_2_ substrate, and Polyimide film are shown in Fig. 2(b,e), respectively. These results indicate the transferring and pattern can be achieved simultaneously by LDW technology. It can be necessary to point out that phase change of the donor material has not been appeared during the process of transferring. The transfer method also can be used to transfer stacks of different materials, especially interesting for the fabrication of multi-layered devices^32^.

As for the preparing of Ag/Bi_2_Se_3_ composition nanofilm, the glass loaded AgNO_3_ film and Bi_2_Se_3_ film is placed in the way of face-to-face and assure to contact closely. The laser beam pass the glass substrate loaded AgNO_3_, AgNO_3_ film, and Bi_2_Se_3_ film in order, and then throughout from the glass substrate loaded Bi_2_Se_3_. AgNO_3_ film absorbs laser energy and transforms into localized heat, leading to the formation of Ag NPs. At the same time, Ag NPs can be sputtered and deposited on the surface of Bi_2_Se_3_ film due to the instantaneous high temperature under the laser action, which gives rise to formation of Ag/Bi_2_Se_3_ composite film. It can be necessary to point out that the intensity of laser and scan rate have great impact on patterning integrality of Ag NPs transferred to the glass loaded Bi_2_Se_3_ film. When the intensities of laser beam is lower than a certain value or the scan speed is too fast, which can result in nanomaterials not transferred or incompletely transferred, even glass substrate can be damaged.

**Figure 3:**
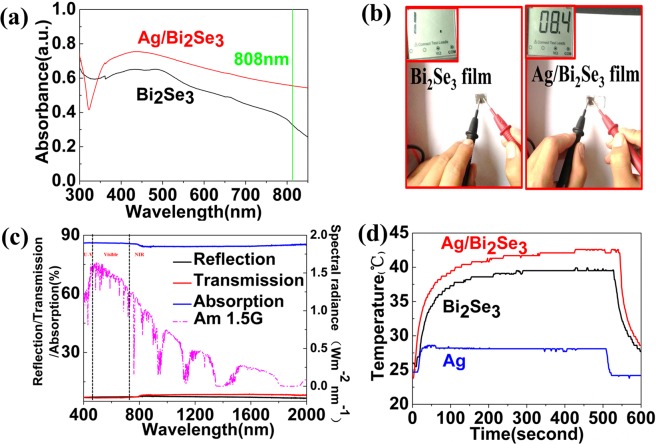
(**a**) UV-vis absorption spectrum of Bi_2_Se_3_ and Ag/Bi_2_Se_3_ films. (**b**) The conductive performance of the Ag NPs based Ag/Bi_2_Se_3_ films. (**c**) Optical properties of Ag/Bi_2_Se_3_ film in the wavelength range of 250−2200 nm, dashed lines represent the spectral solar irradiance density of air mass 1.5 global (AM 1.5 G) tilt solar spectrum. (**d**) Photothermal conversion effect of Ag, Bi_2_Se_3_ and Ag/Bi_2_Se_3_ films.

To demonstrate formation of the composite structure and photothermal characteristic of the sample, measurement results of optical and thermal properties are shown in Fig. 3. The UV-vis-NIR absorbance spectra of Ag/Bi_2_Se_3_ nanocomposite film are shown in Fig. 3(a). It can be clearly seen that there is a broadened absorbance from 500 nm to 800 nm. The UV absorption of the films can be caused by the excitation of electrons from the band-to-band or band-defect transitions^33,34^. The weak absorption near 808 nm is caused by the LSPR in the near infrared band, which is in good agreement with recent theoretical work for Bi_2_Se_3_ nanosheets based on tight-binding theory^35^. An apparent absorption from 350 nm to 400 nm appears for Ag/Bi_2_Se_3_ nanocomposite owing to the surface plasma excitation of spatially confined electrons in nano-sized Ag^0^ particles^36^. The absorption band is considerable broad and red-shifted owing to the different Ag particle size^37^, which can result in large shifts of the emission peak excited by the different wavelengths light. It is also inferred that the Ag nanoparticles indeed grow on the surface of Bi_2_Se_3_ nanosheets. Meanwhile, Ag NPs existing on the surface of Bi_2_Se_3_ can account for the high electrical conductivity, proving by resistance value of multimeter in Fig. 3(b), while none is displayed in Bi_2_Se_3_ film. The result further demonstrates that Ag can grow on the surface of Bi_2_Se_3_ film by LDW technology.

In order to further illuminate the light absorption ability, the experimental measurement of transmission, reflection and absorption spectra for Ag/Bi_2_Se_3_ film is investigated by using a spectrophotometer system with an integrating sphere. As shown in Fig. 3(c), the film exhibits a considerable negligible transmission and low reflection within the limits of spectrum of 400–2200 nm, while corresponding to strong absorption, which can be ascribed to the combined characteristic between broads absorption of the Ag NPs, Bi_2_Se_3_ layer as well as the light scattering of Ag NPs surface, increasing the optical path length in the composite film^38^. It is worth mentioning, the improved surface roughness can enhance the multi-scattering of incident light, benefiting for the effective absorption. In generally, the strong spectrum absorption ability indicates that the Ag/Bi_2_Se_3_ film is a promising photothermal conversion material. Ag/Bi_2_Se_3_ has been proven to be effective in the aspect of photothermal conversion. Figure 3(d) is curves of variances films in temperature including Ag, Bi_2_Se_3_ and Ag/Bi_2_Se_3_ film. It is clearly seen that temperature of Ag/Bi_2_Se_3_ film exhibits rather higher than the other films (Bi_2_Se_3_ and Ag) under irradiation of 1 W laser. The infrared thermometer with accuracy of ±0.1 °C is applied to test the temperature, recording one time per 1 s. The temperature of Ag/Bi_2_Se_3_ composite film rises to balance rapidly within 120 s, while Bi_2_Se_3_ film is in 180 s. And, the temperature of Ag film only increases by 4 °C in Fig. 3(d), it can resist rapid heat dissipation.

**Figure 4:**
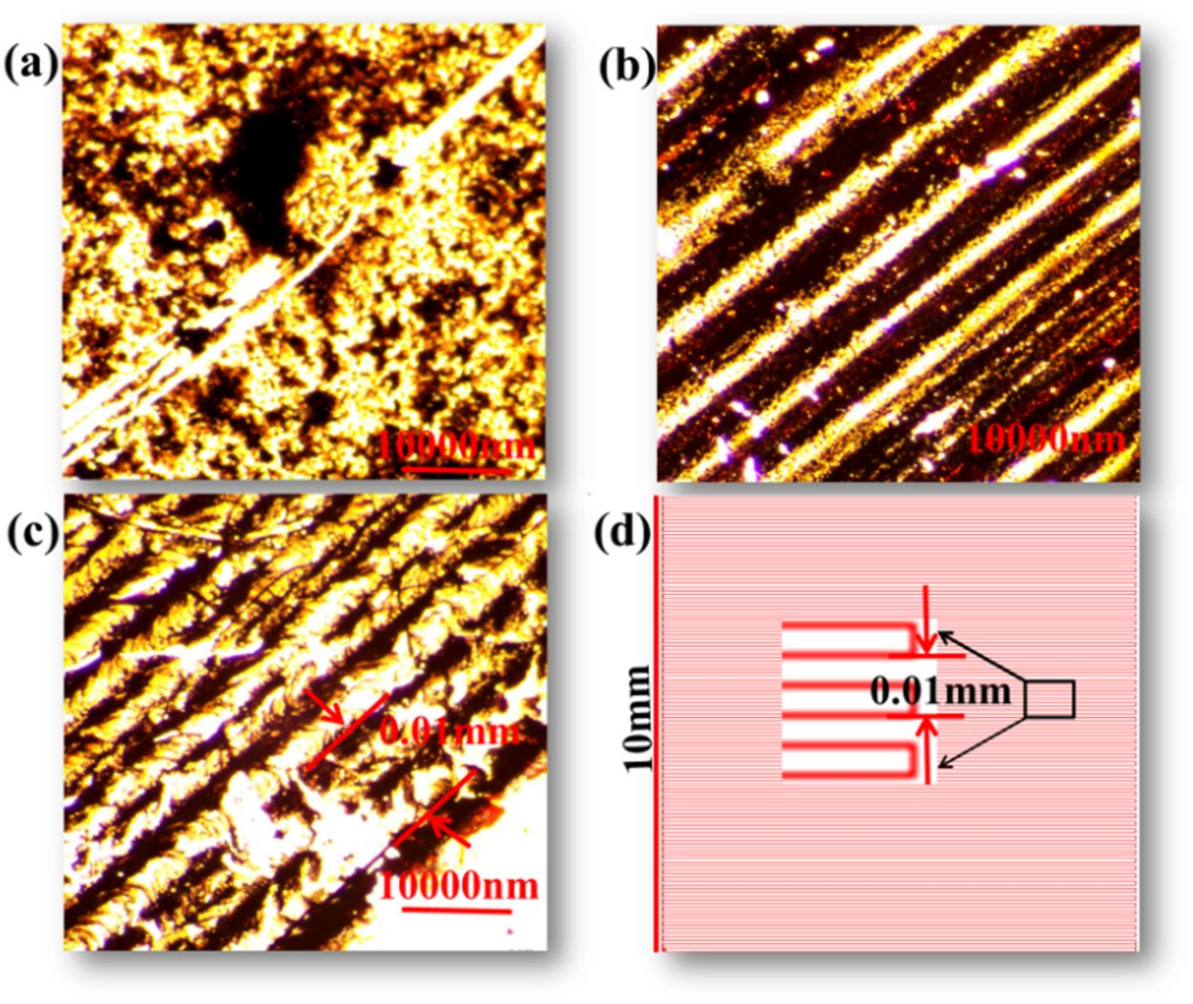
(**a**) Bi_2_Se_3_ film structure. (**b**) A pattern of Ag. (**c**) A pattern of Ag/Bi_2_Se_3_. (**d**) The picture of the path of the laser direct-writing.

As shown in Fig. 4, it is clearly seen that pattern of Bi_2_Se_3_ and Ag/Bi_2_Se_3_ is preform by optical microscope image. Figure 4(a) is optical microscope image of Bi_2_Se_3_ film prepared on a glass slice via the evaporation-induced self-assembly process in Bi_2_Se_3_ nanosheets alcohol solution. The image shows that the highly uniform Bi_2_Se_3_ film can be fabricated based on the self-deposition method. Figure 4(b) show the optical microscope image of Ag pattern on the glass substrate after AgNO_3_ film is acted by the laser. It can be clearly seen that the Ag NPs are formed continually along the track of laser. The interval in Ag pattern transferred to the surface of Bi_2_Se_3_ film is 0.01 mm (Fig. 4(c)), which is well agreement with the set value of laser path in computer (in Fig. 4(d)).

## Discussion

**Figure 5:**
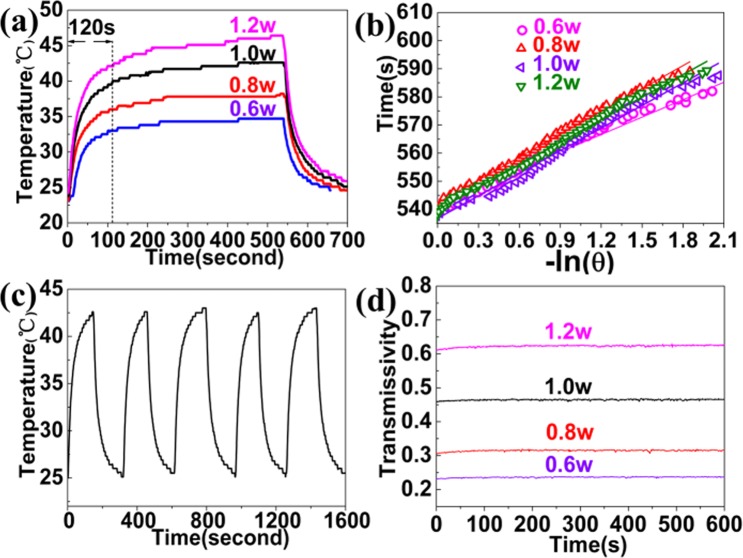
(**a**) Photothermal conversion effect of Ag/Bi_2_Se_3_. (**b**) Time constants with various for heat transfer by fitting. (**c**) Temperature evolution of Ag/Bi_2_Se_3_ composite film. (**d**) Transmissivity of Ag/Bi_2_Se_3_ composite film.

The photothermal conversion image of Ag/Bi_2_Se_3_ composite films are further investigated in near infrared region (Fig. 5). The temperatures of the composite film show remarkable increase within 120 s under irradiation of 808 nm laser. The temperature of Ag/Bi_2_Se_3_ composite film rises rapidly with the increasing of the laser power, eventually reach to 47 °C within 120 s at the laser power in 1.2w, as shown in Fig. 5(a). The final equilibrium temperatures of Ag/Bi_2_Se_3_ nanofilm is increased by around 34.7 °C at 0.6w, 38.2 °C at 0.8w, 42.6 °C at 1.0w and 45.6 °C at 1.2w, respectively. Presently, many thin films, such as Zinc oxide (ZnO), Titanium dioxide (TiO_2_), and aluminum-doped zinc oxide are investigated in the application of photothermal conversion^39–42^. Although they have performed an excellent photothermal conversion effect, which is still essential to improve photothermal conversion capacity. Compared with the previous researching results, the final equilibrium temperature of Ag/Bi_2_Se_3_ nanofilm of is previously higher the reported value. These appearances clearly indicate that Ag/Bi_2_Se_3_ composite film possesses the outstanding capacity of photothermal conversion, which can be considered as a promising photothermal material. The thermal equilibrium time constant can effectively evaluate the heat storage capacity, and can be determined by heat transfer equation^43^. The thermal equilibrium time constants of Ag/Bi_2_Se_3_ composite film with different laser power are obtained for thermal equilibration with the surroundings via conductive and irradiative heat transfer^11^. Figure 5(b) shows a time constant for heat transfer time determined as the negative reciprocal slope of ln (θ) vs. t using temperature versus time data recorded during cooling of the solution. The thermal equilibrium time constant of the samples are calculated to be 20.67, 28.40, 28.07 and 28.10 s for the laser powers 0.6, 0.8, 1.0 and 1.2 s, respectively. It can clearly be seen that these heat transfer time constants remain basically stable with increasing of laser power. The result is attributed to the equilibrium of heat generation and transfer to environment and demonstrating an excellent heat storage capacity of Ag/Bi_2_Se_3_ composite film. Figure 5(c) reveals the temperature elevation cycle performance of Ag/Bi_2_Se_3_ composite film over four laser ON/OFF cycles of laser irradiation. No significant decrease for the temperature elevation is observed for these samples, indicating excellent thermal stability of the Ag/Bi_2_Se_3_ composite film. It can be demonstrated that Ag/Bi_2_Se_3_ composite film are dominant heat sources. Transmissivity of Ag/Bi_2_Se_3_ composite film with various laser powers is presented in Fig. 5(d) It can clearly be seen that transmissivity of various laser powers for Ag/Bi_2_Se_3_ composite film is a horizontal line, revealing the high stability of the film.

**Figure 6:**
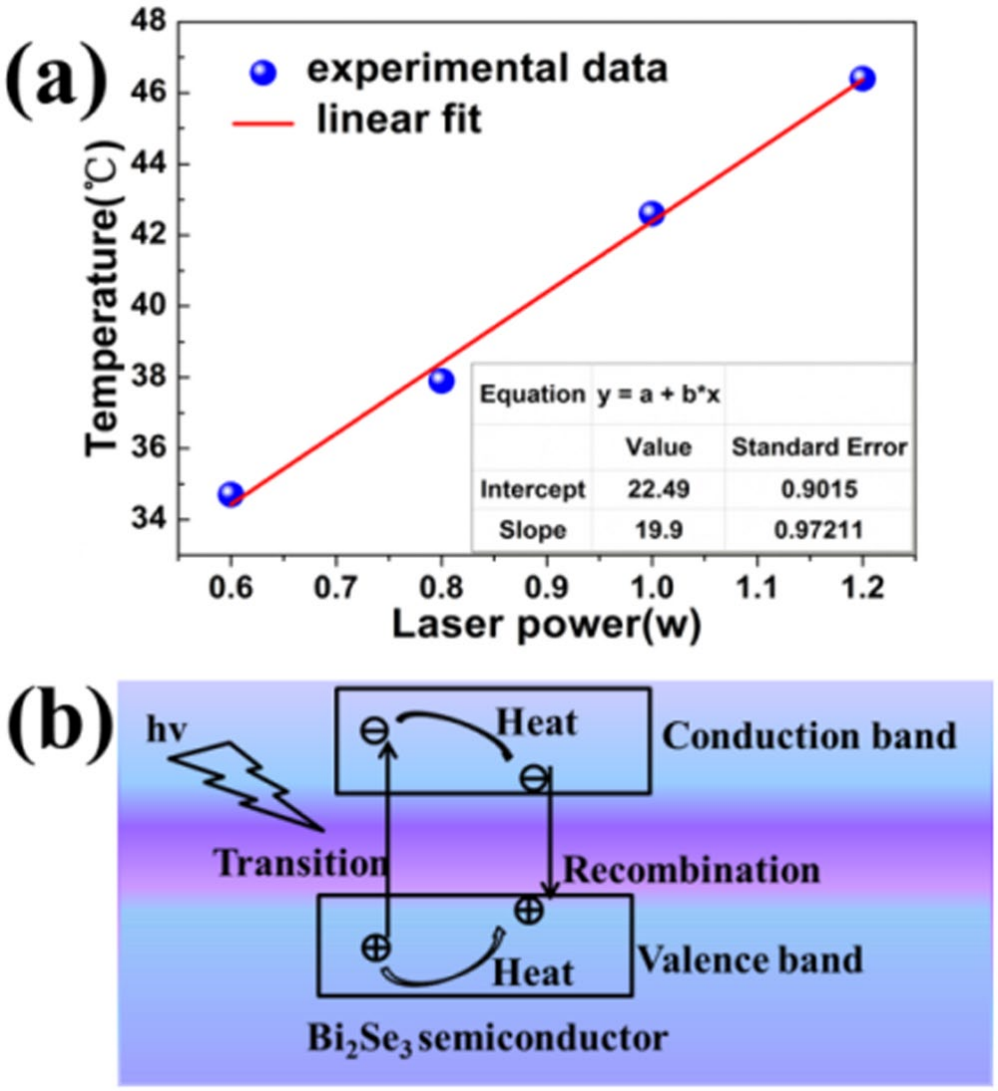
(**a**) Maximum temperature of the sample with increasing laser power. (**b**) Illustration of the electron–hole generation and relaxation in narrow-band gap Bi_2_Se_3_.

Mechanism of Ag/Bi_2_Se_3_ film photothermal conversion is essential to analysis in Fig. 6. Ag/Bi_2_Se_3_ composite film exhibits better photothermal conversion performance than Bi_2_Se_3_ film. The synergistic effect between the Ag NPs with the Bi_2_Se_3_ nanosheets can play an important role in the process of Ag/Bi_2_Se_3_ composite film photothermal conversion. The changing of the Ag/Bi_2_Se_3_ film steady-state temperature is collected as a function of intensity of the light-source (laser power 0.6, 0.8, 1.0 and 1.2w, respectively), and is shown in Fig. 6(a). The intensity vs temperature increases in a linear manner y(x) = 19.9*x + 22.49, indicating that the photothermal effect in the Ag/Bi_2_Se_3_ composite film is intrinsic. This result indicates the photothermal conversion should arise from material itself rather than due to any other factors. The strong absorption of Ag/Bi_2_Se_3_ composite film becomes potential candidates for application of photothermal conversion. It has already been demonstrated that a metal-island film can enhance the absorption, which support LSPR that efficiently couple incident light into the waveguide modes^39^. The guided modes trap the incident light in the active region, enhancing the absorption in a manner analogous to light trapping by surface texturing^44^.

In this case, the incident photon trapped into the Bi_2_Se_3_ film with the help of Ag NPs and enhanced the light absorption. The increasing optical path length and photon coupling process inside the Bi_2_Se_3_ in consequence of Ag NPs plasmonic oscillation, which can lead to the intensive absorption of Bi_2_Se_3_ in visible region. In addition, Ag NPs existing in Bi_2_Se_3_ film cause synergetic effect of multi-scattering induced light trapping, the reflection in interface and the optimal trade-off between light absorption and phonon emission. The process can result in sufficient absorption of incident light. Furthermore, it is worth noting that Ag/Bi_2_Se_3_ film will undergo multiple laser absorption, and a great deal of photons generated under the condition of 808 nm laser irradiation due to LSPR effect. Electrons can be motivated into conduction band while holes still retain in the valence band. The above-bandgap electrons and holes relax to the band edges. The behaviour is called thermalization process, which can convert the excess energy into heat (Fig. 6(b))^43^. The surplus band edge energy is responsible for heat generation and the heat can be absorbed by the vibration of crystal lattice.

In summary, Bi_2_Se_3_ can be transferred on different material substrates by LDW technology, and a composite structure Ag/Bi_2_Se_3_ nanofilm can also be fabricated. The localized heat induced by laser direct achieve simultaneously material synthesis, transfer and patterning in a single processing step. The thermal reaction process includes the forming of nanoparticles based on the process of the thermal reduction, laser ablation, and sputtering deposition and so on. The photothermal measurement experiments show that the composite film possess excellent heat storage capacity and heat stability. The strong absorption can mainly originate from broad absorption of the Ag/Bi_2_Se_3_ layer and the LSPR enhancing of Ag nanoparticles, which can be main factor to result in Ag/Bi_2_Se_3_ composite film owning the excellent photothermal conversion properties.

## Methods

All chemicals were analytical grade and used without further purification. In a typical synthesis of Bi_2_Se_3_ nanosheets, Bi_2_Se_3_ nanosheets are prepared by a one-pot microwave-assisted method. In brief, the synthesis process of Bi_2_Se_3_ is described as follow, 1.1 mmol Na_2_SeO_3_ powder (0.2014g), 4 mmol PVP powers (0.4666 g), 0.8 mmol Bi(NO_3_)_3_·5H_2_O (0.3970 g) and 6.2 mmol NaOH (0.2495 g) are added into ethylene glycol (60 mL), with well-distributed mixing. The mixing solution is heated by the microwave means for 60 min. Then, the solution is cooled down to the room temperature and centrifuged at the speed of 8000 rpm for 30 min, washed three times with deionized water and alcohol, respectively. The powder is collected and dried overnight for further research.

### LDW treatment

As-prepared Bi_2_Se_3_ powder is dispersed in 50 ml alcohol. A uniform Bi_2_Se_3_ thin film can be prepared on the glass substrate by evaporating self-assembling process^45^. The intrinsic glass substrate has been carefully cleaned in a sonicating bath warm acetone (50 °C) for an hour to remove the organic pollutant. The glass slice with self-assembling Bi_2_Se_3_ film is placed face-to-face on the different substrates including silicon, glass, Si/SiO_2_ and Polyimide (PI) and so on. The laser beam with an output power 1100 mW, a wavelength of 470 nm and light spot diameter 100 μm, is focused on Bi_2_Se_3_ films and is scanned at a speed of 1.6 mm/sec. Following laser treatment, the vacant part for Bi_2_Se_3_ films is transferring to various substrates.

The preparing process of AgNO_3_ solution is illustrated as follow. 0.05 mmol AgNO_3_ (0.01 g) power and 0.2 mmol PVP (0.02 g) are dissolved in 0.5 mL alcohol; the mixture is stirred for 10 min. The mixture deposits onto glass substrate to formation of AgNO_3_ film by evaporating self-assembling process, which is similar to the preparing of Bi_2_Se_3_ thin film. The surface of AgNO_3_ film closely contact surface of the as-prepared Bi_2_Se_3_ film, and the laser beam is focused on the AgNO_3_ film and move along the path according to the computer designing. Subsequently, patterning of Ag is carefully transferred to Bi_2_Se_3_ film, resulting in the formation of Ag/Bi_2_Se_3_ composite structure.

### Characterization

UV-vis absorption spectrum is obtained using a Perkin Lambda UV-vis-NIR spectrophotometer. The transparency and reflectance are recorded using a UV-via NIR spectrometer with an integrating sphere unit. X-ray diffraction (XRD) is recorded using a Shimadzu XRD-7000 with Cu-Kradiation. The morphology and characterizations of the obtained samples is assessed on a field emission scanning electron microscope (FESEM, FEI Quanta 200 F) and with transmission electron microscopy (TEM, FEI Tecnai G2 S-Twin) with an operating voltage of 300 kV. Electrical conductivity is measured by multimeter. Optical microscope image is recorded on Leica DM500. All optical measurements are performed at room temperature.

The external adjustable power 808 nm NIR laser with a spot size of 0.6 cm^2^ is used to measure the photothermal conversion performance of nanofilm. The output power is independently calibrated using a handy optical power meter. The *in-situ* temperature is recorded by an infrared thermometer with accuracy of ±0.1 °C tilted 45°relative to the path of the laser.

## References

[1] Volz S, Shiomi J, Nomura M, Miyazaki K (2016). Heat conduction in nanostructured materials Journal of Thermal. Sci. Technol..

[2] Juangsa FB, Muroya Y, Ryu M, Morikawa J, Nozaki T (2017). Comparative study of thermal conductivity in crystalline and amorphous nanocomposite. Appl. Phys. Lett..

[3] Sun ZB, Xie HH, Tang SY (2015). Ultrasmall Black Phosphorus Quantum Dots: Synthesis and Use as Photothermal Agents. Angew chem int edit.

[4] Xing C, Jing G, Liang X (2017). Graphene oxide/black phosphorus nanoflake aerogels with robust thermo-stability and significantly enhanced photothermal properties in air. Nanoscale..

[5] Zijlstra P, Chon JWM, Gu M (2009). Five-dimensional optical recording mediated by surface plasmons in gold nanorods. Nature..

[6] Pustovalov VK (2016). Light-to-heat conversion and heating of single nanoparticles, their assemblies, and the surrounding medium under laser pulses. Rsc. Adv..

[7] Tao W, Ji X, Xu X (2017). Inside Cover: Antimonene Quantum Dots: Synthesis and Application as Near‐Infrared Photothermal Agents for Effective Cancer Therapy. Angew Chem Int Ed Engl..

[8] Neumann O, Urban AS (2013). Solar vapor generation enabled by nanoparticles. Acs Nano..

[9] John SK, John D, Bijoy N, Chathanathodi R, Anappara AA (2017). Magnesium diboride: An effective light-to-heat conversion material in solid-state. Appl. Phys. Lett..

[10] You C (2017). A strategy for photothermal conversion of polymeric nanoparticles by polyaniline for smart control of targeted drug delivery. Nanotechnol..

[11] Jia G, Zhang Y, Wang P (2016). Nano-photo-thermal energy drives MoS_2_/ZnO nanoheterojunctions growing. Opt. Mater. Express..

[12] Jia GZ (2015). Excellent photothermal conversion of core/shell CdSe/Bi_2_Se_3_ quantum dots. Nano Res..

[13] Liu C (2016). Topological insulator Bi_2_Se_3_ nanowire/Si heterostructure photodetectors with ultrahigh responsivity and broadband response. J. Mater. Chem. C..

[14] Cao Y (2013). Mapping the orbital wavefunction of the surface states in three-dimensional topological insulators. Nat. Phys..

[15] Xie H, Li Z, Sun Z (2016). Metabolizable Ultrathin Bi_2_Se_3_ Nanosheets in Imaging-Guided Photothermal Therapy. Small..

[16] Xie H (2017). Near-infrared optical performances of two Bi_2_Se_3_ nanosheets. Rsc Advances.

[17] Liu M (2014). Dual-Wavelength Harmonically Mode-Locked Fiber Laser With Topological Insulator Saturable Absorbe. IEEE Photonic tech l..

[18] Pesin D, MacDonald AH (2012). Spintronics and pseudospintronics in graphene and topological insulators. Nat mater..

[19] Wang ZT (2012). Switchable Dual-Wavelength Synchronously Q-Switched Erbium-Doped Fiber Laser Based on Graphene Saturable Absorber. IEEE Photonics J.

[20] Ying Y, Yang Y, Ying W (2016). Two-dimensional materials for novel liquid separation membranes. Nanotech..

[21] Pathak TK, Swart HC, Kroon RE (2018). Structural and plasmonic properties of noble metal doped ZnO nanomaterials. Physica B..

[22] McFarland AD, Van Duyne RP (2003). Single silver nanoparticles as real-time optical sensors with zeptomole sensitivity. Nano Lett..

[23] Shi J (2014). A tumor-targeting near-infrared laser-triggered drug delivery system based on GO@Ag nanoparticles for chemo-photothermal therapy and X-ray imaging. Biomater..

[24] Sakr GB, Yahia IS, El-Komy GM, Salem AM (2011). Optical properties of thermally evaporated Bi_2_Se_3_ thin films treated with AgNO_3_ solution. Surf. Coat. Technol..

[25] Yang JY, Chen RG, Fan XA, Bao SQ, Zhu W (2006). Thermoelectric properties of silver-doped n-type Bi_2_Te_3_-based material prepared by mechanical alloying and subsequent hot pressing. J. Alloys Compd..

[26] Zacharatos F, Karvounis P, Theodorakos I (2018). Single Step Laser Transfer and Laser Curing of Ag NanoWires: A Digital Process for the Fabrication of Flexible and Transparent, Microelectrodesm. Mater..

[27] Rigoni F (2017). Transfer of CVD-grown graphene for room temperature gas sensors. Nanotechnol..

[28] Oh J-S (2013). Laser-Assisted Simultaneous Patterning and Transferring of Graphene. J. Phys. Chem. C..

[29] In JB, Lee D, Fornasiero F, Noy A, Grigoropoulos CP (2012). Laser-Assisted Simultaneous Transfer and Patterning of Vertically Aligned Carbon Nanotube Arrays on Polymer Substrates for Flexible Devices. Acs Nano..

[30] Zhang Y (2017). Size effect on near infrared photothermal conversion properties of liquid-exfoliated MoS_2_ and MoSe_2_. Superlattices Microstruct.

[31] Zhou K-G, Mao N-N, Wang H-X, Peng Y, Zhang H-L (2011). A Mixed-Solvent Strategy for Efficient Exfoliation of Inorganic Graphene, Analogues Angew. Chem.-Int. Edit..

[32] Stewart JS, Lippert T, Nagel M, Nuesch F, Wokaun A (2012). Red-green-blue polymer light-emitting diode pixels printed by optimized laser-induced forward transfer. Appl. Phys. Lett..

[33] Li G (2011). Preparation and photoelectrochemical performance of Ag/graphene/TiO_2_ composite film. Appl. Surf. Sci..

[34] Liu BS (2005). Photocatalytic mechanism of TiO2-CeO2 films prepared by magnetron sputtering under UV and visible light. Surf. Sci..

[35] Vargas A (2014). The Changing Colors of a Quantum-Confined Topological Insulator. Acs Nano..

[36] Ko S, Banerjee CK, Sankar J (2011). Photochemical synthesis and photocatalytic activity in simulated solar light of nanosized Ag doped TiO_2_ nanoparticle compositeComposites. Part B..

[37] Yu B, Leung KM, Guo Q, Lau WM, Yang J (2011). Synthesis of Ag-TiO_2_ composite nano thin film for antimicrobial application. Nanotechnol..

[38] Ganguly A, Mondal A, Dhar JC, Singh NK, Choudhury S (2013). Enhanced visible light absorption by TiO_2_ film patterned with Ag nanoparticles arrays. Physica E..

[39] Sahu DR, Lin S-Y, Huang J-L (2006). ZnO/Ag/ZnO multilayer films for the application of a very low resistance transparent electrode. Appl Surf Sci..

[40] Wang J, Shi D (2017). Spectral selective and photothermal nano structured thin films for energy efficient windows. Applied Energy..

[41] Kulczyk-Malecka J (2014). Investigation of silver diffusion in TiO_2_/Ag/TiO_2_ coatings. Acta Mater..

[42] Ando E, Miyazaki M (2008). Durability of doped zinc oxide/silver/doped zinc oxide low emissivity coatings in humid environment. Thin Solid Films..

[43] Wang J (2017). High-Performance Photothermal Conversion of Narrow-Bandgap Ti_2_O_3_ Nanoparticles. Adv. Mater..

[44] Jeong S (2012). Hybrid Silicon Nanocone-Polymer Solar Cells. Nano Lett..

[45] Jia G, Wu Z, Wang P, Yao J, Chang K (2016). Morphological evolution of self-deposition Bi_2_Se_3_ nanosheets by oxygen plasma treatment. Sci. Rep..

